# Consequences of interrupted follow-up in head and neck cancer and oral potentially malignant disorders patients due to the COVID-19 pandemic

**DOI:** 10.4317/medoral.27561

**Published:** 2025-10-17

**Authors:** Ana Carolina Evangelista Colafemina, Brenda Corrêa Santos, Beatriz Cristina Cabeza, Ivan Jose Correia-Neto, Isabel Schausltz Pereira Faustino, Alan Roger Santos-Silva, Pablo Agustin Vargas, Márcio Ajudarte Lopes

**Affiliations:** 1 Department of Oral Diagnosis, Piracicaba Dental School, University of Campinas, Piracicaba, São Paulo, Brazil; 2 Department of Stomatology, Public Health and Forensic Dentistry, School of Dentistry of Ribeirão Preto, University of São Paulo, Ribeirão Preto, Brazil

## Abstract

**Background:**

The COVID-19 pandemic caused major disruptions in healthcare services, especially affecting routine monitoring of patients with head and neck cancer (HNC) and oral potentially malignant disorders (OPMDs). Its disruption during the pandemic may have led to delayed diagnoses and worse outcomes.

**Material and Methods:**

This study included patients previously under follow-up at a referral center. All were invited for reassessment involving clinical examination and comparison with pre-pandemic records. Cases were categorized as stable, progressive, regressive (for OPMDs), or deceased. A biopsy was performed on any suspicious changes. Statistical analyses included chi-square, Fisher's exact test, odds ratios, and Kaplan-Meier survival estimates.

**Results:**

A total of 246 patients were evaluated, including 103 patients with HNC and 143 with OPMDs. Patients were re-assessed post-pandemic and again during the latest follow-up. Among HNC, progression was more frequent in the post-pandemic period (26.9%) compared to the later appointment (24%), which was statistically significant (p=0.0011). Stability after the pandemic was linked to better prognosis (OR=4.667; p=0.0051). Survival analysis revealed significantly lower survival rates for the post-pandemic group compared to those followed during subsequent follow-up (HR=0.26; p&lt;0.0001). For OPMDs, progression was observed in 27.28% post-pandemic and 19.14% in the other group (p=0.0214). However, prognosis in the latest follow-up was not significantly associated with post-pandemic status (p=0.1292). Outcome distribution by lesion subtype was significant over time (p=0.0046) and at the latest follow-up (p=0.0088). Patients with proliferative verrucous leukoplakia exhibited a higher rate of progression. Survival analysis also revealed a significantly lower survival rate in the first period (HR=0.52; p=0.037).

**Conclusions:**

These results underscore the lasting consequences of follow-up interruption. Delays in monitoring may lead to progression or delayed diagnosis, especially in high-risk patients.

## Introduction

Head and neck squamous cell carcinoma is the sixth most common malignant tumor worldwide (1). Risk factors for this condition include tobacco and alcohol, as well as human papillomavirus (HPV) infection, particularly HPV-16, which is associated with oropharynx cancer (2). In cases involving lips, chronic exposure to ultraviolet radiation is also recognized as a significant risk factor (2).

Oral potentially malignant disorders (OPMDs), such as leukoplakia, erythroplakia, oral lichen planus (LP), and proliferative verrucous leukoplakia (PVL), represent a group of lesions that are often seen as precursors for oral squamous cell carcinoma (OSCC) (3). Although the risk of malignant transformation varies by subtype and individual risk profile, no reliable biomarkers are currently available to predict malignant transformation (4). Regular clinical follow-up is essential to detect malignant changes early and improve outcomes in these patients. Five-year survival rates for HNC vary depending on stage at diagnosis, ranging from approximately 86.6% for localized disease to 39.3% for metastatic cases (1).

In March 2020, the World Health Organization (WHO) declared the Coronavirus Disease 2019 (COVID-19) a global pandemic. As a result, non-essential health services, including oral diagnostics and oncology, were suspended. This interruption affected the clinical follow-up of patients with HNC, delaying diagnoses that could have been made earlier (5). Some studies reported a 70% reduction in oral diagnostic procedures in referral centers, which probably contributed to worse prognoses in many cases (6).

Recent studies have shown that patients diagnosed during the pandemic often presented with more advanced disease stages and poorer outcomes compared to pre-pandemic cases (7 , 8). Delays in treatment, caused by resource limitations and overloaded hospitals, also contributed to the lower survival rates seen in HNC patients (9). In oral medicine, reduced dental visits compromised early diagnosis and management of OPMDs, possibly increasing the risk of malignant transformation (10).

Given this scenario, our study focused on a group of patients with HNC and OPMDs who were being followed before the pandemic. They were evaluated after services resumed and again at a later follow-up. We aimed to evaluate disease progression, changes in lesions, and clinical outcomes over time. This investigation provides insights into the consequences of healthcare service interruptions on cancer monitoring and early detection in this high-risk population.

## Material and Methods

Study design

This was an observational, longitudinal study conducted at a referral center for oral medicine. We included two groups of patients: Individuals diagnosed with malignant tumors and those with OPMDs. All patients had been under regular follow-up at the institution before the COVID-19 pandemic.

Clinical examination and follow-up

All patients were invited to attend a follow-up consultation. During the appointment, a standardized protocol was used, including both extraoral and intraoral examination under adequate lighting. Assessments were performed by two experienced clinicians: An oral medicine specialist and a professor with over 30 years of expertise in diagnosing and managing oral lesions.

Each evaluation was documented through photographic records. These photographs, combined with detailed notes in the patients' medical charts, allowed for comparisons over time regarding lesion size, surface, color, and texture.

In patients with a history of HNC, signs of recurrence at the primary site, new lesion development, lymph node involvement, and evidence of systemic progression were assessed. For OPMD cases, we compared the current clinical presentation with the most recent pre-pandemic records, considering factors such as lesion size, alterations in surface morphology, color changes, thickness, and the emergence of new lesions.

If any suspicious clinical changes were noted, such as alteration in morphology, ulceration, increased infiltration, or suspicious features indicating malignant transformation, a biopsy was performed to confirm the diagnosis. Based on clinical progression, cases were classified as stable, progressive (recurrence, metastasis, or malignant transformation), regressive (for OPMDs), or deceased.

The interval between the post-pandemic appointment and the most recent follow-up varied across patients. On average, this period was 28.06 months, ranging from 3 to 40 months, depending on clinical availability and the timing of patient return.

Demographic and clinical data were extracted from medical records, including age, gender, lesion location, histopathological diagnosis, and treatments performed. Treatment modalities for malignant tumors included surgery, radiotherapy, chemotherapy, or combined modalities.

Statistical analysis

Statistical analyses were performed using R software (version 4.3.1) and GraphPad Prism (version 10.4.2). For the associations between clinical outcomes and categorical variables (such as lesion type or follow-up year), we used the chi-square test or Fisher's exact test, depending on the distribution and sample size. For larger contingency tables with multiple categories, we applied Fisher's exact test with Monte Carlo simulation to ensure accurate estimation of significance. We also calculated relative risks (RR) and odds ratios (OR) to quantify the strength of association between the patients' initial clinical status and their outcomes during follow-up. For survival data, Kaplan-Meier estimates were generated, and differences between groups were compared using the Cox proportional hazards regression model. These analyses were conducted using the survival and survminer packages in R. Additionally, multivariate regression analyses were performed to identify independent prognostic factors. Covariates included gender, age, smoking, alcohol status, and lesion-related variables. Statistical significance was indicated as *p&lt;0.05, **p&lt;0.01, ***p&lt;0.001, and ****p&lt;0.0001.

## Results

A total of 408 patients were initially under clinical follow-up, including 178 diagnosed with HNC and 230 with OPMDs. Among those with HNC, 103 patients (57.9%) returned for follow-up after the pandemic, including 68 men (66%) and 35 women (34%), with a mean age of 65.3 years. The most affected sites were the tongue (31;30.1%), floor of the mouth (16;15.5%), lips (8;7.8%), and larynx (6;5.8%). All other tumor sites together accounted for 30 cases (29.1%). Five patients (4.9%) had tumors involving more than one anatomical site (Table 1).

Table 1

Histologically, OSCC was the predominant type, identified in 91 cases (88.3%), followed by mucoepidermoid carcinoma (3;2.9%), lymphoma (3;2.9%), adenoid cystic carcinoma (2;2%), carcinoma ex-pleomorphic adenoma (1;1%), polymorphous low-grade adenocarcinoma (1;1%), and melanoma (1;1%). Regarding treatment, 79 patients (76.7%) underwent surgery, 36 (35%) received radiotherapy, and 26 (25.2%) received a combination of radiotherapy and chemotherapy. Treatment information was unavailable for 36 patients (23.2%) (Table 1).

At the post-pandemic evaluation, 64 of the 103 patients (62.1%) were clinically stable, while 39 (37.9%) showed signs of disease progression. This included local recurrence in 10 cases (25.6%), metastasis in 2 (5.1%), and new primary tumors in 4 (10.3%). In the follow-up conducted later, among the 68 patients who returned, 50 (73.5%) remained stable, while 18 (26.5%) had progressed, with 7 recorded deaths.

**Figure 1:**
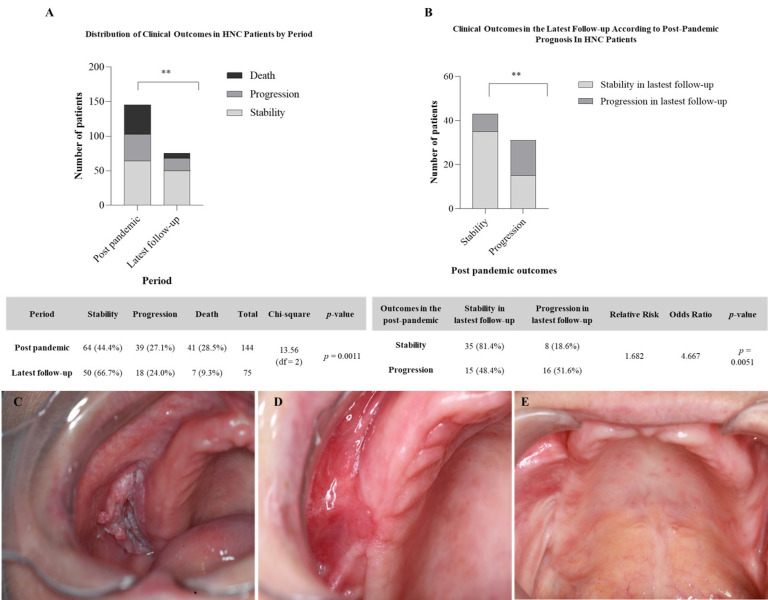
Clinical outcomes in HNC patients. (A) Distribution of outcomes (stability, progression, death) in the post-pandemic period vs. the latestfollow-up (p=0.0011, Chi-square). (B) Prognosis in the latest follow-up according to post-pandemic status. Patients stable after the pandemic weremore likely to remain stable (RR=1.68; OR=4.67; p=0.0051) (C) Initial diagnosis of SCC on the superior vestibular fornix before the pandemic, followedby treatment (D) Post-pandemic follow-up revealing development of a new lesion diagnosed as severe epithelial dysplasia (E) Latest follow-upshowing absence of recurrence after treatment.

Of the 39 patients who experienced post-pandemic disease progression, 31 attended a later follow-up. Among them, 15 (60%) showed clinical stabilization. Of these, 15 (60%) showed clinical stabilization, 10 (40%) remained in progression, and 6 had died. Among the 64 patients who were initially stable, 43 returned in the latest follow-up: 35 (72%) remained stable, 7 (14%) progressed, and 1 died. The comparison between the two periods revealed a statistically significant difference (Chi-square=13.56; p=0.0011), indicating that the pandemic negatively affected the clinical course of patients with malignant tumors (Figure 1A).

1

A prognostic analysis confirmed the importance of early follow-up based on post-pandemic clinical status. Patients stable at the first evaluation were more likely to remain stable over time. The relative risk of maintaining stability was 1.76, and the odds ratio (OR=3.06; p=0.042) confirmed the association between early stability and favorable outcomes (Figure 1B).

This pattern is illustrated in Figure 1C-E, which show the clinical progression of a patient who was initially diagnosed with SCC on the superior vestibular fornix prior to the pandemic (Figure 1C). During the pandemic period, the patient developed a new lesion that was diagnosed as severe epithelial dysplasia (Figure 1D). After treatment, the patient did not present recurrence at the most recent follow-up (Figure 1E).

Clinical outcomes varied according to the histopathological type of HNC. Most complications, including progression and death, occurred in patients with SCC. Adenoid cystic carcinoma and lymphomas also showed some unfavorable outcomes. In contrast, no deaths were observed in rare subtypes such as melanoma and polymorphous adenocarcinoma (Table 2).

Table 2

Among patients with SCC, clinical outcomes varied according to the tumor site. The tongue was the most affected region and showed the highest number of progressions and deaths during the post-pandemic period. Other sites with notable negative outcomes included the floor of the mouth and oropharynx. In contrast, regions such as the palate, buccal mucosa, and alveolar ridge had fewer cases of progression or death (Table 3).

Table 3

A total of 143 patients (62.2%) from the OPMDs group returned after the pandemic, with 53 men (37.1%) and 90 women (62.9%), and a mean age of 65.33 years. The most common type of lesion was lichen planus (52 cases;36.4%), followed by leukoplakia (51 cases;35.7%), proliferative verrucous leukoplakia (22 cases;15.4%), lichenoid reaction (4 cases;2.8%), and erythroplakia (1 case;0.7%) (Table 4).

Table 4

Of the 143 patients assessed after the pandemic, 34 (23.8%) showed lesion regression, 70 (48.9%) remained stable, and 39 (27.3%) progressed. In the latest follow-up, among the 94 who returned, most remained stable (63 patients;67%), while 18 (19.2%) progressed and 13 (13.8%) regressed. Two patients who progressed developed squamous cell carcinoma and died.

**Figure 2:**
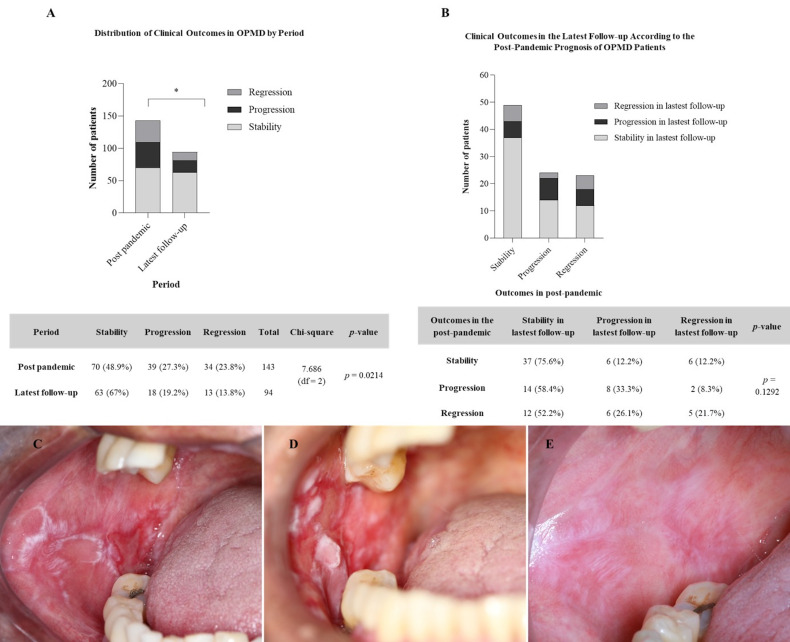
Clinical outcomes in patients with OPMDs. (A) Distribution of clinical outcomes (stability, progression, regression) in thepost-pandemic period and the latest follow-up (Chi-square=7.686; p=0.0214). (B) Clinical outcomes in the latest follow-up based on postpandemicstatus (p=0.1292). (C) Initial presentation observed in the pre-pandemic period. (D) First post-pandemic consultation showingpersistence and thickening of the lesion. (E) Most recent follow-up revealing significant clinical regression.

Of the 39 patients who had shown progression after the pandemic, 24 returned later: 8 (33.3%) were still in progression, 14 (63.6%) were stable, and 2 (9.1%) showed improvement. Of the 70 initially stable patients, 49 returned, with 37 (75.6%) maintaining stability, 6 (12.2%) improving, and 6 (12.2%) progressing. Finally, among the 34 patients who initially improved, 23 returned: 12 (52.2%) remained stable, 6 (26.1%) progressed and 5 (21.7%) showed further regression. A statistically significant difference was observed between the post-pandemic and past evaluation clinical outcomes (Chi-square=7.68; p=0.0214), suggesting that the pandemic also significantly influenced the course of potentially malignant disorders (Figure 2A).

2

However, no statistically significant association was found between the clinical status in the post-pandemic period and the latest follow-up outcomes (p=0.1292). While stable patients in post-pandemic were more likely to remain stable (75.5%) and those who had progressed tended to follow a worse course, these differences did not reach statistical significance. Individuals who had initially improved showed mixed results, with 52.2% remaining stable and only 21.7% continuing to regress (Figure 2B).

An example of this clinical variability is shown in Figure 2C-E. The patient initially presented a lesion before the pandemic (Figure 2C), which worsened at the first post-pandemic visit (Figure 2D), but showed significant regression at the latest follow-up (Figure 2E).

**Figure 3:**
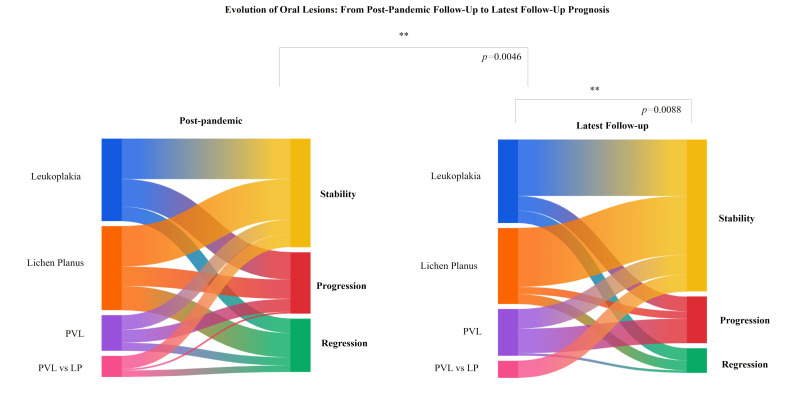
Evolution of OPMDs by lesion subtype over time. The diagram shows the clinical outcomes (stability, progression,regression) of OPMD by subtype at post-pandemic and in the latest follow-up. Outcome distribution variedsignificantly by lesion type at both time points (p=0.0046 and p=0.0088, respectively).

Stratifying outcomes by lesion subtype provided additional insights. A statistically significant difference in outcome distribution across lesion types between post-pandemic and latest follow-up (p=0.0046), suggesting changes in behavior over time. Furthermore, lesion subtype remained a significant prognostic factor later (p=0.0088), reinforcing the relevance of lesion classification in long-term follow-up (Figure 3).

3

**Figure 4:**
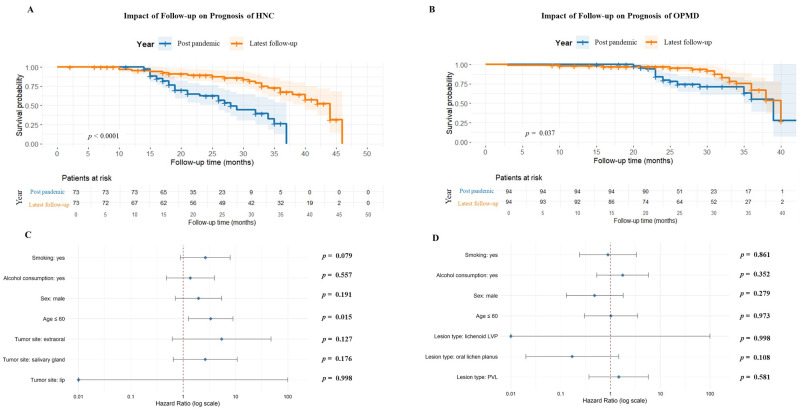
Survival analysis of patients according to follow-up period. (A) HNC patients followed in the post-pandemic period had significantly lower survival probabilities compared to those evaluated inthe latest follow-up (HR=0.26; 95% CI: 0.14-0.48; p&lt;0.0001). (B) Similarly, patients with OPMDs showed worse survival in the post-pandemic period compared to the other group (HR=0.52, 95% CI: 0.28-0.97; p=0.037). (C) Multivariate Cox regression for HNC identified age ≤60 years as a significant predictor of worse prognosis; other variables were not statistically significant. (D) In the OPMD group, noclinical variables were significantly associated with prognosis.

Survival analysis showed a significant difference in prognosis according to the follow-up year. For patients with HNC, those followed in the post-pandemic period had lower survival probabilities compared to those followed in the latest follow-up, with the difference being highly significant (HR=0.26; 95% CI: 0.14-0.48; p&lt;0.0001) (Figure 4A). A similar pattern was observed among patients with oral potentially malignant disorders, where survival was also significantly worse for the post-pandemic cohort compared to the later follow-up group (HR=0.52, 95% CI: 0.28-0.97; p=0.037) (Figure 4B). Multivariate analysis for HNC identified age 60 years as a significant predictor of worse prognosis (HR: 3.36; p=0.015), while the other variables were not statistically associated with outcomes (Figure 4C). In the OPMD group, none of the evaluated variables showed significant associations with prognosis (Figure 4D).

4

## Figures and Tables

**Table 1:** Table Clinical and demographic characteristics of patients with HNC.

Characteristics	Number of cases (n=103)
Age	
Mean	65.3
Gender	
Male	68
Female	35
Location	
Tongue	31
Floor of the mouth	16
Lip	8
Salivary gland	8
Alveolar ridge	6
Buccal mucosa	6
Larynx	6
Palate	4
Oropharynx	4
Others	18
Histology	
Squamous cell carcinoma	91
Mucoepidermoid	3
Lymphoma	3
Adenoid cystic carcinoma	2
Carcinoma ex-pleomorphic adenoma	1
Polymorphous adenocarcinoma	1
Melanoma	1
Treatment	
Surgery	79
Radiotherapy	36
Chemoradiotherapy	26
Chemotherapy	3

1

**Table 2:** Table Clinical outcomes of patients with HNC according to histopathologicaltype.

Type of HNC	Stability	Progression	Death	Loss to follow-up	Total cases
SCC					
Post-pandemic	56	36	38	27	157
Last follow-up	46	14	7	25	
Adenoid cystic carcinoma					
Post-pandemic	1	1	0	2	4
Last follow-up	1	1	0	0	
Mucoepidermoid					
Post-pandemic	2	1	0	1	4
Last follow-up	0	1	0	2	
B-cell non-Hodgkin lymphoma					
Post-pandemic	2	0	1	1	4
Last follow-up	1	0	0	1	
Hodgkin lymphoma					
Post-pandemic	0	0	1	1	2
Last follow-up	0	0	0	0	
Carcinoma ex-pleomorphic adenoma					
Post-pandemic	1	0	0	1	2
Last follow-up	1	0	0	0	
Burkitt lymphoma					
Post-pandemic	1	0	0	0	1
Last follow-up	0	0	0	1	
Melanoma					
Post-pandemic	1	0	0	0	1
Last follow-up	1	0	0	0	
Polymorphous adenocarcinoma					
Post-pandemic	0	1	0	0	1
Last follow-up	0	1	0	0	
Others					
Post-pandemic	0	0	1	1	2
Last follow-up	0	1	0	0	

2

**Table 3:** Table Clinical outcomes of patients with SCC according to theanatomical site of the tumor, comparing the post-pandemic periodand the latest follow-up.

Site of SCC	Stability	Progression	Death
Tongue			
Post-pandemic	14	17	15
Last follow-up	14	6	3
Floor of the mouth			
Post-pandemic	11	5	6
Last follow-up	4	3	1
Lip			
Post-pandemic	6	2	3
Last follow-up	6	0	0
Oropharynx			
Post-pandemic	2	2	3
Last follow-up	0	1	2
Larynx			
Post-pandemic	6	0	2
Last follow-up	1	1	0
Alveolar ridge			
Post-pandemic	3	3	2
Last follow-up	5	0	0
Palate			
Post-pandemic	2	2	1
Last follow-up	2	2	0
Buccal mucosa			
Post-pandemic	3	3	1
Last follow-up	3	1	1
Other locations			
Post-pandemic	9	6	6
Last follow-up	11	1	2

3

**Table 4:** Table Clinical and demographic characteristics of patientswith OPMD.

Characteristics	Number of cases (n=143)
Age	
Mean	65.33
Gender	
Male	53
Female	90
Lesion	
Lichen Planus	52
Leukoplakia	51
PVL	22
PVL vs LP	13
Lichenoid Reaction	4
Erythroplakia	1

4

## Data Availability

The datasets used and/or analyzed during the current study are available from the corresponding author.
